# The Fixed-Dose Combination of Losartan/Hydrochlorothiazide Elicits Potent Blood Pressure Lowering During Nighttime in Obese Hypertensive Patients

**DOI:** 10.4021/jocmr1649w

**Published:** 2013-12-13

**Authors:** Chikao Ibuki, Yoshihiko Seino, Toshiaki Otsuka, Kyoichi Mizuno

**Affiliations:** a Cardiovascular Center, Nippon Medical School Chiba Hokusoh Hospital, Chiba, Japan; b Department of Hygiene and Public Health, Nippon Medical School, Tokyo, Japan; c Division of Cardiology, Nippon Medical School, Tokyo, Japan

**Keywords:** Fixed-dose combination, Antihypertensive agent, Angiotensin receptor blocker, Nocturnal blood pressure, Obesity, Thiazide, Ambulatory blood pressure monitoring

## Abstract

**Background:**

Hypertension is one of the most powerful predictor of the future cardiovascular events, and antihypertensive therapy adopting multiple drug regimen is often needed to obtain the appropriate blood pressure (BP) control. To clarify the blood pressure-lowering effect of the fixed-dose combination (FDC) of an angiotensin receptor blocker (ARB) and diuretic agent in poorly controlled hypertensive patients, we intended a multicenter prospective observational study (Investigation for Normalized Blood pressure control with the Appropriate medication: INBA) by means of the sequential ambulatory blood pressure monitoring (ABPM).

**Methods:**

One hundred and thirteen hypertensive patients who had not achieved the target BP control proposed in the guidelines with medication containing any ARB but without diuretic agents (54 men; mean age, 66 years old; mean office systolic/diastolic BP (SBP/DBP), 158/82 mmHg) were enrolled. Daytime and nighttime blood pressures were assessed with ABPM before and at 12 weeks after switching the ARB to the FDC of 50 mg of losartan, and 12.5 mg of hydrochlorothiazide (HCTZ).

**Results:**

Daytime SBP/DBP (mean ± SD) decreased from 151 ± 14/88 ± 8 mmHg to 140 ± 11/82 ± 8 mmHg (P < 0.001, P < 0.001, respectively), and nocturnal SBP/DBP from 138 ± 18/78 ± 9 mmHg to 125 ± 14/72 ± 9 mmHg (P < 0.001, P < 0.001, respectively) during the 12 weeks treatment. Pulse rate did not change irrespective of the time window. Among various parameters (age, history of hypertension, body mass index (BMI), serum potassium, uric acid, estimated glomerular filtration rate, plasma B-type natriuretic peptide), BMI alone showed significant negative correlation with 12-weeks reduction in nocturnal SBP (r = -0.43, P < 0.05). No parameters correlated with reduction in daytime SBP during this period. Patients with BMI ≥ median (25.8 kg/m^2^) showed significantly greater reduction in nocturnal SBP for 12 weeks than patients with BMI < median (20.1 ± 15.6 mmHg vs 6.1 ± 10.9 mmHg, P < 0.001) although reduction in daytime SBP was comparable between the two groups (8.9 ± 13.5 mmHg vs 11.9 ± 12.7 mmHg).

**Conclusions:**

The administration of the FDC of losartan/HCTZ lowers BP both in day- and nighttime, and the nocturnal antihypertensive potency is remarkable in obese patients.

## Introduction

Hypertension is an important predictive or predisposing factor for future cardiovascular (CV) events [1, 2]. Guidelines have proposed the target blood pressure (BP) according to the patient’s risk profile, and the combination therapy with 2 or more antihypertensive agents is recommended when the target BP cannot be achieved with single use of either agent [3-5]. For better adherence to the antihypertensive drug regimen, the fixed-dose combination (FDC) of angiotensin receptor blocker (ARB) either with diuretic or calcium channel blocker (CCB) has been recently introduced.

The out-of-office BP is superior to the office BP to predict CV events in hypertensive patients and general population [6-8], and especially the nighttime BP level determined by ambulatory blood pressure monitoring (ABPM) is associated with target organ damage and its regression [9]. However, how the ARB-based FDC affects the circadian BP variation in hypertensive patients in the real-world practice remains to be determined, and few studies have investigated what kind of clinical factors contribute to the BP-lowering effect.

To clarify the BP-lowering effect of the FDC of 50 mg of losartan, an ARB, and low dose (12.5 mg) hydrochlorothiazide (HCTZ), we intended a multicenter prospective observational study (Investigation for Normalized Blood pressure control with the Appropriate medication: INBA), in which the circadian BP of hypertensive outpatients was sequentially evaluated by means of ABPM in the real-world practice.

## Materials and Methods

This study was conducted by Hokusoh Hypertension Study Group, composed with 20 general physicians’ clinics in the Hokusoh community of Chiba prefecture Japan, from May 2008 to August 2010, and was approved by the Investigation Review Board of the Osaka Foundation for the Prevention of Cancer and Cardiovascular Diseases. All patients gave written informed consent for their participation.

### Study population

Outpatient subjects with hypertension, who had not achieved the target BP proposed by the guideline of Japanese Society of Hypertension (JSH 2004) [10] despite antihypertensive medication for more than 4 weeks with any ARB but without diuretics, were enrolled. Exclusion criteria were followings; Age < 20 or ≥ 80 years; malignant hypertension or diastolic BP (DBP) ≥ 110 mmHg; active (NYHA III or more) heart failure; severely impaired renal (serum creatinine ≥ 1.5 mg/dL) and/or liver function; recent (within 6 months) myocardial infarction; definite or probable pregnancy.

### Protocol

In all patients, the ARB which had been prescribed was switched to the FDC of 50 mg of losartan and 12.5 mg of HCTZ. Patients were instructed to take the FDC tablet once a day in the morning. Before and at 12 weeks after the commencement of the FDC, 24-hour ABPM was performed. Left ventricular ejection fraction was assessed with echocardiography at baseline period, and left ventricular hypertrophy was estimated with ECG according to the modified Sokolow-Lyon criteria [11]. Biochemical laboratory data (serum potassium, creatinine, and uric acid) and plasma B-type natriuretic peptide (BNP) were assessed before and at 12 weeks of the study. At each measurement, blood samples were obtained intravenously after overnight fasting. The estimated glomerular filtration rate (eGFR) was calculated according to the following equation presented by the Japanese Society of Nephrology [12]; eGFR (mL/min/1.73m^2^) = 194 × serum creatinine^-1.094^ × age^-0.287^ (× 0.739, if female).

### ABPM

In each time, the ABPM device (TM 2030, A and D, Saitama, Japan) was equipped in the morning on a weekday and systolic/diastolic BP (SBP/DBP), and pulse rate were recorded by means of the oscillometric method every 30 minutes (6 am - 12 pm) or 60 minutes (12 pm - 6 am) for 24 hours. All ABPM data were analyzed at Nippon Medical School Chiba Hokusoh Hospital. Among all ABPM data, the first-hour measurement was not included in the analysis. The nighttime data was defined as the average of recordings while the patient stayed in the bed, based on the patient’s behavior report, and the daytime data defined as the average of the remaining period. Nocturnal BP dipping (%) was defined as ((daytime SBP-nighttime SBP)/daytime SBP). Nocturnal minimum SBP and morning SBP were defined according to the method adopted by Kario et al [13]; Nocturnal minimum SBP: the average of 3 consecutive recordings centered on the lowest SBP during the nighttime. Morning SBP: the average of SBP data during the first 2 hours after leaving bed.

### Statistical analysis

Numerical values were expressed as the mean ± standard deviation. Serial changes in the ABPM parameters and laboratory data were assessed with paired t-test or the Wilcoxon test. The correlation between BP parameter changes during the 12-weeks study period and clinical parameters was analyzed with Spearman’s test. Comparison of the BP reduction between subgroups stratified by body mass index (BMI) was made with unpaired t-test. Values of P < 0.05 were regarded as being statistically significant.

## Results

The baseline characteristics of the study subjects are shown in Table 1. The mean age was 66.0 years and 54% of subjects were male. Nearly half patients showed left ventricular hypertrophy on ECG. The most prevalent antihypertensive agent besides ARB was calcium channel blocker (CCB).

**Table 1:** Baseline Characteristics of the Study Patients

Gender (male %)	54 (50%)
Office BP, mmHg	
systolic	158 ± 12
diastolic	82 ± 8
Diabetes (%)	20 (18%)
Age, years	66.0 ± 9.7
Smoking (%)	27 (25%)
History of hypertension, years	6.5 ± 4.8
Body mass index	26.4 ± 3.7
Left ventricular hypertrophy on ECG (%)	54 (50%)
Left ventricular ejection fraction, %	69 ± 6
BNP, pg/mL	60 ± 61
eGFR, mL/min/1.73m^2^	73 ± 23
Co-prescription	
Calcium channel blocker (%)	64 (59%)
Statin (%)	35 (32%)
Previous ARB	
Losartan	14
Valsartan	36
Candesartan	26
Telmisartan	22
Olmesartan	15

BP: blood pressure; BNP: B-type natriuretic peptide; eGFR: estimated glomerular filtration rate; ARB: angiotensin receptor blocker.

**Figure 1:**
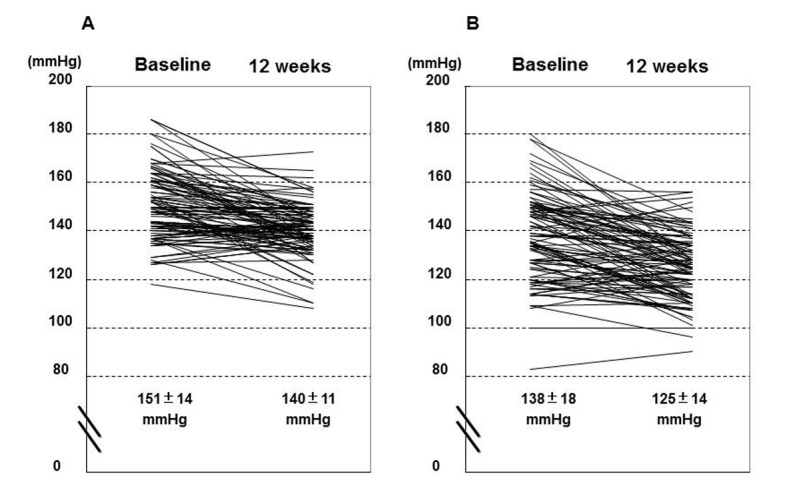
Individual plot of mean systolic blood pressure (SBP) at baseline and 12 weeks after the commencement of fixed-dose combination of losartan/hydrochlorothiazide; (A), daytime; (B), nighttime.

Successful recordings of ABPM both at baseline and 12 weeks were obtained in 109 patients. Both of day- and nighttime BP parameters significantly decreased after the 12 weeks treatment (Table 2) (Fig. 1). Pulse rate did not change irrespective in the day and night time period. Systolic and diastolic BP reduction in the daytime after the switching to the FDC were 11.0 ± 13.7 mmHg and 5.7 ± 7.3 mmHg, respectively, and these values were comparable to the BP reduction in the nighttime (13.1 ± 15.0 mmHg and 5.8 ± 8.6 mmHg, respectively). At baseline, nocturnal SBP was lower than the daytime (8.5 ± 11.0%), and this dipping was preserved even at 12 weeks after (10.8 ± 8.8%). The minimum SBP during the nighttime and morning SBP significantly reduced after the 12 weeks treatment.

**Table 2:** Sequential Change of Ambulatory Blood Pressure Monitoring (ABPM) Parameters and Laboratory Data

	Baseline	12 weeks	P
ABPM parameters;			
Daytime			
SBP, mmHg	151 ± 14	140 ± 11	P < 0.001
DBP, mmHg	88 ± 8	82 ± 8	P < 0.001
Pulse rate, /min	70 ± 7	71 ± 9	NS
Nighttime			
SBP, mmHg	138 ± 18	125 ± 14	P < 0.001
DBP, mmHg	78 ± 9	72 ± 9	P < 0.001
Pulse rate, /min	60 ± 8	61 ± 8	NS
Nocturnal SBP dipping, %	8.5 ± 11	10.8 ± 8.8	P < 0.1
Nocturnal minimum SBP, mmHg	134 ± 18	117 ± 15	P < 0.001
Morning SBP, mmHg	156 ± 20	144 ± 15	P < 0.001
Laboratory data			
Serum potassium, mEq/L	4.3 ± 0.4	4.1 ± 0.4	P < 0.001
Serum UA, mg/dL	5.7 ± 1.1	5.6 ± 0.9	NS
eGFR, ml/min/1.73m^2^	73 ± 23	69 ± 18	P < 0.1
Plasma BNP, pg/mL	60 ± 61	38 ± 30	P < 0.001

SBP, systolic blood pressure; DBP, diastolic blood pressure; UA, uric acid; eGFR, estimated glomerular filtration rate; BNP, B-type natriuretic peptide.

**Figure 2:**
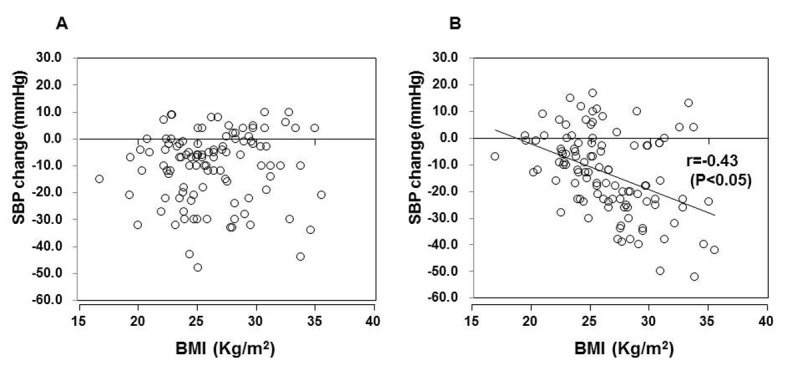
Correlation of sequential change of systolic blood pressure (SBP) in the daytime (A) and nighttime (B) during 12 weeks of the study and baseline body mass index (BMI). A statistically significant correlation was observed between the nocturnal SBP change and BMI (r = -0.43, P < 0.05).

**Figure 3:**
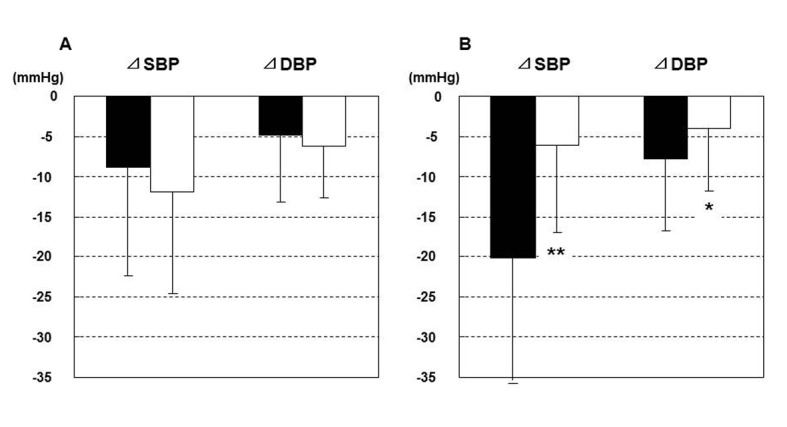
Change of systolic blood pressure (SBP) in the daytime (A) and nighttime (B) during 12 weeks. Closed bar indicates patients with body mass index (BMI) ≥ 25.8 kg/m^2^, and open bar patients with BMI < 25.8 kg/m^2^; *, P < 0.05 and **, P < 0.001 between the high and low BMI subgroups.

Among various parameters (age, history of hypertension, BMI, serum potassium, uric acid, eGFR, plasma BNP), only BMI showed the significant negative correlation with 12-weeks reduction in nocturnal SBP (Fig. 2). Patients with BMI more than or equal to the median (25.8 kg/m^2^) showed significantly greater reduction in SBP during nighttime than patients with BMI < 25.8 (20.1 ± 15.6 mmHg vs 6.1 ± 10.9 mmHg, P < 0.001) (Fig. 3). Reduction in daytime SBP was, however, comparable between these two groups (8.9 ± 13.5 mmHg vs 11.9 ± 12.7 mmHg).

In whole patients, there was no serious clinical event during the present study. With regard to the laboratory data, serum potassium significantly decreased after the 12 weeks treatment (Table 2) although this change was minor and within the physiological range. BNP significantly decreased after the 12 weeks treatment, however there was no significant change in uric acid and eGFR.

## Discussion

In the present study, the administration of FDC of losartan/HCTZ lowered BP both in day- and nighttime in poorly controlled hypertensive patients treated with an ARB. Plasma BNP level decreased significantly during the study period, and there was no clinically meaningful change in serum electrolytes and uric acid levels, nor the renal function. Interestingly, BMI showed significant negative correlation with nocturnal SBP reduction. Hypertension is one of the most prevalent pathological states for general practitioners in outpatients’ care [1]. The present findings provide an important implication for the application of the FDC, that is, this therapeutic regimen would elicit the largest benefit in the certain patient profile, namely obese hypertensive patients.

### Rationale for the combination of renin-angiotensin system (RAS) inhibitor and diuretic

Pharmacological inhibition of RAS adopting angiotensin converting enzyme (ACE) inhibitors or ARB in hypertensive patients exhibits the consistent effects in the prevention of CV/cerebral events [14]. However, it is well known that in the ‘low renin state’ such as elderly people the BP-lowering effect of RAS inhibitors attenuates [15]. Thiazide is an antihypertensive drug that could efficiently lower BP particularly in salt-sensitive patients through the mechanism of natriuresis. In a landmark clinical trial using thiazide diuretic as the only antihypertensive agent, the substantial BP-lowering effect and the prevention of CV events was shown in patients with isolated systolic hypertension [16]. Uzu el al have reported that in diabetic patients the addition of diuretic to an ARB resulted in marked reduction in nocturnal BP compared with the monotherapy with valsartan [17]. It should be pointed out that, in the LIFE (Losartan Intervention For Endpoint reduction) study, a trial that investigated for stroke prevention of losartan, HCTZ was added to achieve proper BP values in more than 70% cases [18], implicating the superiority of combination of losartan/HCTZ but not the single use of losartan to atenolol-based regimen. The present finding that the addition of thiazide remarkably reduced the nocturnal BP in the obese subgroup is consistent with previous reports that diuretic exerted the potent BP-lowering effect in the salt-sensitive state [19].

On the other hand, it has been known that the administration of high dose thiazide diuretic exacerbates the risk of hypokalemia and hyperuricemia [20], despite the substantial BP-lowering effect, and that unfavorable outcomes such as sudden cardiac death might develop as a result of these metabolic derangements [21]. We should notice that thiazide could act as the ‘enhancer’ of antihypertensive power of RAS inhibition through the combination therapy with a RAS inhibitor rather than the monotherapy, and detrimental effects such as electrolytes imbalance and impaired insulin sensitivity can be canceled mutually by the combination. In this point, MacKay et al have indicated that the combination of losartan and HCTZ elicits BP-reduction synergistically through complementary mechanisms [22]. In our study, serum potassium level significantly decreased although this change was minor, and uric acid did not increase. This finding would be mediated via 1) augmented excretion of uric acid by losartan through inhibition of URAT1 [23], and 2) relatively low dose of HCTZ (12.5 mg) adopted in this study. Furthermore, plasma BNP exhibited significant reduction during the present study period, suggesting the attenuation of myocardial stress provided by the antihypertensive regimen with FDC of ARB and thiazide.

### The importance of the nighttime BP in the clinical setting

The noninvasive, automatic BP recording using the ABPM device has enabled the analysis of the circadian variation of BP in hypertensive patients [24]. Among several ABPM parameters, the nighttime or sleep-time BP elicits the more potent predictive power for future CV events compared with 24-hour average or the daytime BP not only in hypertensive patients but also in general population [6, 8]. It has been documented in a prospective trial [7] and the international database [25] that the elevation in nocturnal BP is linearly correlated with the risk of CV diseases. High value of the nighttime BP or attenuated decline of nocturnal BP (nondipper) is also associated with target organ damage such as left ventricular hypertrophy [26], silent cerebral damage [27], and microalbuminuria [28]. Hansen et al have shown through a meta-analysis including 23,856 hypertensive subjects that the hazard ratio associated with each 10-mmHg increase in nocturnal SBP were 1.16 and 1.19 for mortality and CV morbidity, respectively [29]. The etiology of the nocturnal hypertension is multifactorial; volume overload (heart failure and chronic kidney disease (CKD)), dysfunction of autonomic nervous system (diabetes), insulin resistance and augmented salt sensitivity (metabolic syndrome), and documented sleep apnea might be involved in the increase in nocturnal BP [30]. In CKD, sodium excretion during daytime is often diminished, and the nighttime BP is kept high for the compensatory sodium excretion through the pressure-dependent natriuresis [31]. Thus, target organ damage observed in patients with nocturnal hypertension is not merely the consequence of high pressure but the comorbidity (CKD) per se may contribute to nocturnal hypertension.

It has been explored whether the pharmacological intervention to restore the nighttime BP variation improves the prognosis. The HOPE (Heart Outcomes Prevention Evaluation) study has proved the prognostic effect of an ACE-inhibitor ramipril on CV morbidity and mortality in high risk hypertensive patients despite modest changes in office BP (-3/-2 mmHg in average SBP/DBP) [32], although in its ABPM substudy marked reduction in nocturnal BP (-17/-8 mmHg) was observed in ramipril group [33]. Furthermore, Hermida et al prospectively treated 3344 subjects for a median follow-up period of 5.6 years [34]. In that study, analysis of ABPM revealed that a 5-mmHg reduction in nocturnal SBP was associated with significant 17% attenuation in CV events, independent of changes in any other ABPM parameters. The BP reduction by 20 mmHg during nighttime observed in the present study could be translated to substantial benefit to prevent CV events and target organ damage.

### The antihypertensive treatment with FDC regimen

To control BP in hypertensive patients, more than 2 antihypertensive agents of different class are often required. Multi-drug regimen, however, may compromise patients’ adherence to the therapy and inflate socio-economic costs. The meta-analysis of the FDC clearly shows that the compliance significantly increase by 21% compared with 2 separate pills, and the BP-lowering and the incidence of adverse reactions tend to improve [35]. In previous studies that investigate the effects of the FDC of an ARB and thiazide diuretics have shown reduction in the nocturnal BP [36], urinary albumin excretion [37], and plasma BNP [38], all consistent with our findings. However, the present study is, as far as we know, the first report that investigates the association of the BP-lowering effect of the FDC and the patients’ background, namely significant influence of obese.

CCB is another antihypertensive agent recommended in the guidelines, and is one of the most frequently prescribed drug for hypertension. In Japan at present, both types of the ARB-based FDC with diuretic or CCB are clinically available. The ACCOMPLISH (Avoiding Cardiovascular Events through Combination Therapy in Patients Living with Systolic Hypertension) trial has shown that, in high-risk patients with hypertension, the combination of an ACE inhibitor (benazepril) and a CCB (amlodipine) is superior to that of the ACE inhibitor and HCTZ for the prevention of vascular events [39], while in Japan which FDC regimen is better to attenuate the risks of target organ damage and CV events remains to be established. The present study showed that, at least in patients who have a clinical background of obesity, the FDC of the ARB and HCTZ elicited potent BP-lowering during nighttime.

### Limitations of the study

The present study has several limitations. First, this study is of the single arm design with relatively small size. But, the present study showed significant changes in ABPM parameters following the administration of the FDC of losartan/HCTZ, suggesting that this therapeutic regimen is beneficial for patients with poorly controlled hypertension. Which of CCB or thiazide is an appropriate counterpart of ARB to obtain the sufficient antihypertensive efficacy should be clarified in future trials. Second, mortality and morbidity are not evaluated in the present study. It should be emphasized, however, that this study intended to a therapeutic effect by means of ABPM parameters as the surrogate, and the present finding particularly in BP-lowering during nighttime would have a certain significance.

### Conclusions

Switch to the FDC of losartan/HCTZ in poorly controlled hypertensive patients who had been treated with an ARB resulted in significant BP reduction during day- and nighttime. The BP reduction during nighttime was negatively correlated with BMI, and the present regimen would be a favorable therapeutic option for obese patients with poorly controlled hypertension.

## Members of Hokusoh Hypertension Study Group (Participants of the INBA Study)

Takeaki Kuroda, Mitsuo Kataoka, Masatoshi Ikeshita, Akitsugu Furuya, Tsutomu Iwai, Kunihiko Kinjo, Toshiyuki Kobayashi, Masato Tomimura, Makoto Uchida, Mamoru Imai, Kouji Seimiya, Kenichi Tokuyama, Satoyuki Ito, Yuji Ohkubo, Kimimichi Ichimura, Mikio Satoh, Sumio Shikano, Akira Ohta, Yasuichi Amamoto, Hideki Nishida, Hideko Ishibashi.

## References

[1] Lida M, Ueda K, Okayama A, Kodama K, Sawai K, Shibata S, Tanaka S (2003). Impact of elevated blood pressure on mortality from all causes, cardiovascular diseases, heart disease and stroke among Japanese: 14 year follow-up of randomly selected population from Japanese -- Nippon data 80. J Hum Hypertens.

[2] Kannel WB (1995). Framingham study insights into hypertensive risk of cardiovascular disease. Hypertens Res.

[3] Chobanian AV, Bakris GL, Black HR, Cushman WC, Green LA, Izzo JL, Jr., Jones DW (2003). The Seventh Report of the Joint National Committee on Prevention, Detection, Evaluation, and Treatment of High Blood Pressure: the JNC 7 report. JAMA.

[4] Mansia G, De Backer G, Dominiczak A, Cifkova R, Fagard R, Germano G, Grassi G (2007). 2007 ESH-ESC Guidelines for the management of arterial hypertension: the task force for the management of arterial hypertension of the European Society of Hypertension (ESH) and of the European Society of Cardiology (ESC). Blood Press.

[5] Ogihara T, Kikuchi K, Matsuoka H, Fujita T, Higaki J, Horiuchi M, Imai Y (2009). The Japanese Society of Hypertension Guidelines for the Management of Hypertension (JSH 2009). Hypertens Res.

[6] Clement DL, De Buyzere ML, De Bacquer DA, de Leeuw PW, Duprez DA, Fagard RH, Gheeraert PJ (2003). Prognostic value of ambulatory blood-pressure recordings in patients with treated hypertension. N Engl J Med.

[7] Staessen JA, Thijs L, Fagard R, O'Brien ET, Clement D, de Leeuw PW, Mancia G (1999). Predicting cardiovascular risk using conventional vs ambulatory blood pressure in older patients with systolic hypertension. Systolic Hypertension in Europe Trial Investigators. JAMA.

[8] Ohkubo T, Hozawa A, Nagai K, Kikuya M, Tsuji I, Ito S, Satoh H (2000). Prediction of stroke by ambulatory blood pressure monitoring versus screening blood pressure measurements in a general population: the Ohasama study. J Hypertens.

[9] Mancia G, Zanchetti A, Agabiti-Rosei E, Benemio G, De Cesaris R, Fogari R, Pessina A (1997). Ambulatory blood pressure is superior to clinic blood pressure in predicting treatment-induced regression of left ventricular hypertrophy. SAMPLE Study Group. Study on Ambulatory Monitoring of Blood Pressure and Lisinopril Evaluation. Circulation.

[10] (2006). Japanese Society of Hypertension guidelines for the management of hypertension (JSH 2004). Hypertens Res.

[11] Schillaci G, Verdecchia P, Borgioni C, Ciucci A, Guerrieri M, Zampi I, Battistelli M (1994). Improved electrocardiographic diagnosis of left ventricular hypertrophy. Am J Cardiol.

[12] Matsuo S, Imai E, Horio M, Yasuda Y, Tomita K, Nitta K, Yamagata K (2009). Revised equations for estimated GFR from serum creatinine in Japan. Am J Kidney Dis.

[13] Kario K, Pickering TG, Umeda Y, Hoshide S, Hoshide Y, Morinari M, Murata M (2003). Morning surge in blood pressure as a predictor of silent and clinical cerebrovascular disease in elderly hypertensives: a prospective study. Circulation.

[14] Turnbull F, Neal B, Pfeffer M, Kostis J, Algert C, Woodward M, Chalmers J (2007). Blood pressure-dependent and independent effects of agents that inhibit the renin-angiotensin system. J Hypertens.

[15] Waeber B (2003). Combination therapy with ACE inhibitors/angiotensin II receptor antagonists and diuretics in hypertension. Expert Rev Cardiovasc Ther.

[16] (1991). Prevention of stroke by antihypertensive drug treatment in older persons with isolated systolic hypertension. Final results of the Systolic Hypertension in the Elderly Program (SHEP). SHEP Cooperative Research Group. JAMA.

[17] Uzu T, Sakaguchi M, Yokomaku Y, Kume S, Kanasaki M, Isshiki K, Araki S (2009). Effects of high sodium intake and diuretics on the circadian rhythm of blood pressure in type 2 diabetic patients treated with an angiotensin II receptor blocker. Clin Exp Nephrol.

[18] Dahlof B, Devereux RB, Kjeldsen SE, Julius S, Beevers G, de Faire U, Fyhrquist F (2002). Cardiovascular morbidity and mortality in the Losartan Intervention For Endpoint reduction in hypertension study (LIFE): a randomised trial against atenolol. Lancet.

[19] Wenzel UO, Benndorf R, Lange S (2013). Treatment of arterial hypertension in obese patients. Semin Nephrol.

[20] Messerli FH, Makani H, Benjo A, Romero J, Alviar C, Bangalore S (2011). Antihypertensive efficacy of hydrochlorothiazide as evaluated by ambulatory blood pressure monitoring: a meta-analysis of randomized trials. J Am Coll Cardiol.

[21] Franse LV, Pahor M, Di Bari M, Somes GW, Cushman WC, Applegate WB (2000). Hypokalemia associated with diuretic use and cardiovascular events in the Systolic Hypertension in the Elderly Program. Hypertension.

[22] MacKay JH, Arcuri KE, Goldberg AI, Snapinn SM, Sweet CS (1996). Losartan and low-dose hydrochlorothiazide in patients with essential hypertension. A double-blind, placebo-controlled trial of concomitant administration compared with individual components. Arch Intern Med.

[23] Hamada T, Ichida K, Hosoyamada M, Mizuta E, Yanagihara K, Sonoyama K, Sugihara S (2008). Uricosuric action of losartan via the inhibition of urate transporter 1 (URAT 1) in hypertensive patients. Am J Hypertens.

[24] Pickering TG, Shimbo D, Haas D (2006). Ambulatory blood-pressure monitoring. N Engl J Med.

[25] Boggia J, Li Y, Thijs L, Hansen TW, Kikuya M, Bjorklund-Bodegard K, Richart T (2007). Prognostic accuracy of day versus night ambulatory blood pressure: a cohort study. Lancet.

[26] Verdecchia P, Schillaci G, Guerrieri M, Gatteschi C, Benemio G, Boldrini F, Porcellati C (1990). Circadian blood pressure changes and left ventricular hypertrophy in essential hypertension. Circulation.

[27] Shimada K, Kawamoto A, Matsubayashi K, Ozawa T (1990). Silent cerebrovascular disease in the elderly. Correlation with ambulatory pressure. Hypertension.

[28] Bianchi S, Bigazzi R, Baldari G, Sgherri G, Campese VM (1994). Diurnal variations of blood pressure and microalbuminuria in essential hypertension. Am J Hypertens.

[29] Hansen TW, Li Y, Boggia J, Thijs L, Richart T, Staessen JA (2011). Predictive role of the nighttime blood pressure. Hypertension.

[30] Yano Y, Kario K (2012). Nocturnal blood pressure and cardiovascular disease: a review of recent advances. Hypertens Res.

[31] Fukuda M, Kimura G (2012). Salt sensitivity and nondippers in chronic kidney disease. Curr Hypertens Rep.

[32] Yusuf S, Sleight P, Pogue J, Bosch J, Davies R, Dagenais G (2000). Effects of an angiotensin-converting-enzyme inhibitor, ramipril, on cardiovascular events in high-risk patients. The Heart Outcomes Prevention Evaluation Study Investigators. N Engl J Med.

[33] Svensson P, de Faire U, Sleight P, Yusuf S, Ostergren J (2001). Comparative effects of ramipril on ambulatory and office blood pressures: a HOPE Substudy. Hypertension.

[34] Hermida RC, Ayala DE, Mojon A, Fernandez JR (2011). Decreasing sleep-time blood pressure determined by ambulatory monitoring reduces cardiovascular risk. J Am Coll Cardiol.

[35] Gupta AK, Arshad S, Poulter NR (2010). Compliance, safety, and effectiveness of fixed-dose combinations of antihypertensive agents: a meta-analysis. Hypertension.

[36] Minami J, Abe C, Akashiba A, Takahashi T, Kameda T, Ishimitsu T, Matsuoka H (2007). Long-term efficacy of combination therapy with losartan and low-dose hydrochlorothiazide in patients with uncontrolled hypertension. Int Heart J.

[37] Fukutomi M, Hoshide S, Eguchi K, Watanabe T, Shimada K, Kario K (2012). Differential effects of strict blood pressure lowering by losartan/hydrochlorothiazide combination therapy and high-dose amlodipine monotherapy on microalbuminuria: the ALPHABET study. J Am Soc Hypertens.

[38] Hosoya T, Kuriyama S, Ohno I, Kawamura T, Ogura M, Ikeda M, Ishikawa M (2012). Antihypertensive effect of a fixed-dose combination of losartan/hydrochlorothiazide in patients with uncontrolled hypertension: a multicenter study. Clin Exp Nephrol.

[39] Jamerson K, Weber MA, Bakris GL, Dahlof B, Pitt B, Shi V, Hester A (2008). Benazepril plus amlodipine or hydrochlorothiazide for hypertension in high-risk patients. N Engl J Med.

